# Graft-size selection and anisometropia reduction in penetrating keratoplasty (PKP)

**DOI:** 10.1371/journal.pone.0218199

**Published:** 2019-06-20

**Authors:** Ellen H. Koo, William J. Feuer, Richard K. Forster

**Affiliations:** Bascom Palmer Eye Institute, Department of Ophthalmology, Miller School of Medicine at the University of Miami, Miami, Florida, United States of America; Cleveland Clinic, UNITED STATES

## Abstract

**Purpose:**

To compare the amount of myopia induced by same-size donor-to-host penetrating keratoplasty with that of the amount of myopia induced by over-sized donor-to-host penetrating keratoplasty.

**Setting:**

Tertiary referral academic center.

**Design:**

Retrospective cohort study.

**Methods:**

Charts from patients who underwent penetrating keratoplasty by the same technique at Bascom Palmer Eye Institute between Nov 1, 2002, and January 1, 2006, were reviewed. The patients underwent optical penetrating keratoplasty using 12 interrupted 10–0 nylon sutures and a 12-bite continuous 10–0 nylon suture by a single surgeon (R.K.F.). The surgical technique used would be considered standard of care at most institutions. The Institutional Review Board, University of Miami Human Subjects Research Office, approved the study protocol. The donor graft was over-sized by 0.25mm in eyes when the intended final refractive target was greater than -1.00 diopters spherical equivalent (SE). The same-size donor graft was used when the intended final refractive target was less than -1.00 diopters SE. The selection of donor graft size was entirely based upon clinical parameters, meaning that the intended final refractive target was determined per each patient’s fellow eye refraction, with the intention of reducing anisometropia. All patients received postoperative refraction and corneal topography. These measurements were performed at 6–8 weeks when the initial removal of sutures commenced, then at 6 months, then after completion of selective suture removal, then again at 12 months.

**Results:**

At 12 months, the over-sized group resulted in -1.35 diopters (SD = 2.25) SE of refraction, and the same-size group resulted in -0.14 diopters (SD = 2.42) SE. This approached statistical significance (p = 0.052) in comparison to -1.00 diopters spherical equivalent.

**Conclusions:**

Using a donor graft that is over-sized by 0.25mm results in refraction of -1.00 diopters SE or more of myopia. Using a same-size donor-graft results in refraction of less than -1.00 diopters SE. Therefore, careful graft-size selection can result in a more favorable clinical outcome—namely, reduction in anisometropia—in patients undergoing penetrating keratoplasty.

## Introduction

One of the biggest challenges after penetrating keratoplasty (PKP) is the management of the patient’s refractive outcome. To this date, the refractive outcome after PKP still remains variable, even though graft survival rate 3–5 years following penetrating keratoplasty has reached 90%[1–3]. The lack of predictability is a challenge both for clinician and the patient, as a reasonable refractive outcome translates into meaningful vision for the patient.

Anisometropia in particular, decreases the likelihood of binocular visual functioning for the patient, unless the patient chooses to use a contact lens. It was shown previously that modification of suturing technique did not reliably reduce resultant anisometropia[4]. The technique of selective suture removal—where the clinician removes sutures at the steep axis, as determined by topographical imaging and refraction—was shown to induce myopia[5].

The question remains as to whether it is possible to control the refractive outcome at the time of perioperative planning, specifically, the graft-size selection based on the patient’s fellow eye refraction. To our knowledge, ours is the first study to demonstrate that planned reduction of anisometropia can be achieved by graft-size selection during surgery.

## Methods

### Ethics statement

This retrospective cohort study was approved by the Institutional Review Board (IRB)/Ethics Committee at the University of Miami, and was conducted in conformity with the Declaration of Helsinki. Due to the retrospective nature of the study, informed consent was waived by the IRB/Ethics Committee. All data were fully de-identified and made anonymous before being accessed for the study.

None of the transplant donors were from a vulnerable population and all donors or next of kin provided written informed consent that was freely given.

### Inclusion and exclusion criteria

A chart review of all patients who underwent optical penetrating keratoplasty (PKP) by the same surgeon (R.K.F.) using the same technique between Nov 1, 2002, and January 1, 2006 at Bascom Palmer Eye Institute was performed. Data were gathered from 75 eyes from 72 patients, who had undergone optical penetrating keratoplasty (PKP), and met the inclusion criteria. The exclusion criteria for the study were the following: patients with keratoconus (including re-operations), therapeutic grafts, hypotonous eyes, eyes operated with only interrupted sutures, and all lamellar grafts. For the intra-study exclusion criteria, the following cases were excluded: patients with less than 6 months of follow-up, inability to obtain refraction data in the first 6 months, patients lost to follow-up or unable to keep office follow-up at our institution, and patients where the continuous suture became exposed and required removal.

### Surgical technique

All penetrating keratoplasty procedures were performed using 12 interrupted 10–0 nylon sutures and a 12-bite continuous 10–0 nylon suture. We had previously described this technique as an approach to reducing postkeratoplasty myopia, astigmatism and anisometropia[4]. The donor graft size was determined based on the fellow-eye’s refractive status, and consequently the desired refraction of the surgical-eye. The graft was over-sized by 0.25mm when the intended final refraction was -1.00 diopter SE or more. The same-size graft was selected when the intended final refraction was less myopic than -1.00 diopter SE. Both the donor button and the recipient bed were prepared with the Barron-Hessburg vacuum trephine (Katena Products, Inc, Denville, NY). In cases that required a combined intraocular lens placement—such as concurrent cataract surgery, secondary intraocular lens, or lens exchange—the average keratometry values of 46 were used to calculate the intraocular lens power. All these patients received donor grafts that were over-sized by 0.25mm. An exception was made for patients with a hyperopic correction for the fellow eye, in which case a same-size donor graft was used to avoid a myopic result.

### Data collection

All patients received refraction and corneal topography (TMS-2 Tomey, Phoenix, AZ) starting at their postoperative visit at 6–8 weeks. Topography- guided selective suture removal was initiated 6–8 weeks after surgery and sutures inducing more than 3 diopters were removed. More sutures were removed in this fashion upon subsequent visits at 1–3 month intervals, until the residual astigmatism measured was 3 diopters or less. The continuous suture was left intact. The refraction and corneal topography measurements were taken at subsequent visits at 6 months, after completion of suture removal, and again at 12 months.

### Statistical analyses

Continuous data were summarized with means and standard deviations and compared between groups with the two-tailed two-sample t-test. Dichotomous and polychotomous variables were summarized with percentages and compared with Yates corrected chi-squared.

## Results

### Patient baseline characteristics

Out of the 72 patients, we identified 47 females (65%) and 25 males (35%), with a mean age of 73 years (SD = 13). The underlying diagnoses of the included eyes are illustrated in Table 1; they comprised of 44 eyes with pseudophakic corneal edema (59%), 11 cases with corneal decompensation from Fuchs’ Dystrophy (15%), 11 cases with failed grafts requiring repeat penetrating keratoplasty (15%), 7 cases with corneal scarring (9%), and 2 cases with herpetic disease (3%).

**Table 1:** Underlying pre-operative diagnoses.

Diagnoses	Number of Cases	Percentages
Pseudophakic Corneal Edema	44	59%
Fuchs’ Corneal Dystrophy	11	15%
Failed Grafts	11	15%
Corneal Scars	7	9%
Herpetic Disease	2	3%

### Planned reduction of anisometropia

**Fig 1:**
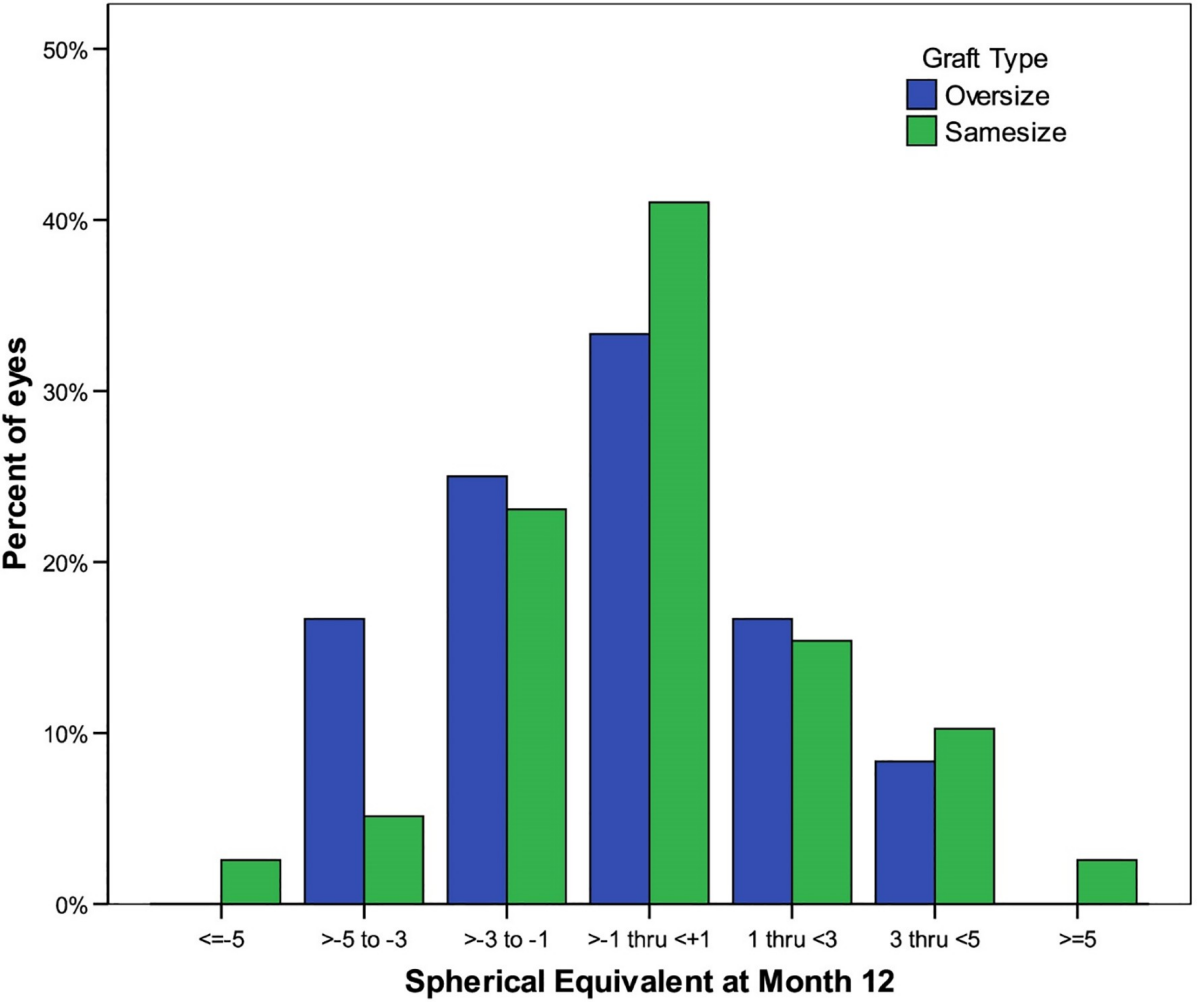
Spherical equivalence in diopters of refraction at 12 months.

At 12 months, 68 out of the 75 eyes met the requirements for the follow-up period; out of these 68 eyes, we considered the 45 eyes with best-corrected visual acuity of 20/50 or better for purposes of our analysis of refractive error. The over-sized group consisting of 13 eyes resulted in an average of -1.35 diopters (± 2.25) spherical equivalent of refraction, p = 0.052 in comparison to plano, and the same-size group consisting of 32 eyes had average -0.14 diopters (± 2.42), p = 0.052) in comparison to -1.00 diopters. The 95% confidence interval around the mean spherical equivalent in the over-sized group extended from -2.7 to 0.0, just including a value of plano, and the 95% confidence interval around the mean spherical equivalent in the same-size group extended from -1.0 to 0.7, just including with a lower limit of -1 diopter. Thus, despite the overlap in the results of the two graft sizing techniques, their distributions are shifted in the desired direction (Fig 1).

## Discussion

Penetrating keratoplasty (PKP) continues to play an important role in visual rehabilitation for patients with corneal opacities. However, a clear cornea is usually insufficient for meaningful visual functioning for the patient. The refractive outcomes—which include myopia, astigmatism and anisometropia—need to be optimized in order to maximize visual functioning. Unwanted extremes in refractive outcomes after penetrating keratoplasty continue to be a challenge in visual rehabilitation after penetrating keratoplasty. Anisometropia needs to be minimized to allow for successful binocular visual functioning for the patient after penetrating keratoplasty.

The elderly subset of patients, are particularly vulnerable to poor visual functioning as a result of undesirable refractive outcomes after PKP. Anisometropia in particular, has been shown to cause disturbances in binocular vision and stereopsis, and is linked to increased falls in the elderly[6–9]. Contact lenses typically help alleviate anisometropia and continue to benefit those who have been fitted successfully. However, not every patient can wear a contact lens. In fact, fitting the elderly patient with a contact lens is considered challenging; factors such as the aging ocular adnexa, increase in dry-eye as well as dry-eye inducing systemic medications, and decline in manual dexterity can lead to contact lens intolerance or non-compliance[10].

More recently, laser-assisted in situ keratomileusis has shown to be useful in correcting myopia and astigmatism following penetrating keratoplasty[11–15]. However, primary penetrating keratoplasty surgical techniques and postoperative suture management have yet to become successful in reliably reducing myopia and anisometropia[4].

In 1980, Cottingham introduced the concept of selective removal of interrupted sutures post penetrating keratoplasty, with the technique of combined interrupted and continuous sutures[16]. Davison and Bourne demonstrated an average astigmatism of 3.6 diopters after removal of the 10–0 nylon continuous suture, using the double running suture technique[17]. Others demonstrated efforts to address the possibility of reducing astigmatism and myopia, usually with either adjustment of the continuous suture, or with selective removal of interrupted sutures[16–22]. However, the question remains as to whether adjusting the surgical technique, in particular, controlling donor-graft size selection, could minimize anisometropia.

Based on our earliest study, we had shown that the techniques of combined interrupted and continuous 10–0 nylon with selective removal of interrupted sutures, provided 75% of patients with vision of 20/50 or better at one year[5]. Thus, this surgical technique of combined interrupted and continuous 10–0 nylon was used for this study. The same study had also demonstrated that selective removal of interrupted sutures reduced astigmatism; however, myopic shift was induced with increasing number of interrupted sutures removed[5]. This was theorized to be due to the increase in corneal curvature seen with the increase in the number of interrupted sutures removed. Our subsequent study showed that placing a tighter continuous suture, with an increase in the average keratometry values to 46.00 diopters for lens calculation in cases of concurrent intraocular placement, along with a lesser-aggressive selective suture removal (meaning more interrupted sutures were left in place), would lead to reduced postkeratoplasty myopia, while maintaining acceptable astigmatism[4]. Based on these findings, for this study, we used the average keratometry values of 46.00 diopters for calculations in cases with combined intraocular lens placement.

Our study demonstrates that planned reduction of anisometropia can be achieved by donor graft size selection based on the fellow’s eye’s refraction, in order to allow for early visual rehabilitation. At 12 months, the over-sized group resulted in -1.35 diopters SE, which trends toward statistical significance (p = 0.052) in comparison to plano. At 12 months, the same-size group resulted in -0.14 diopters SE, which approaches statistical significance (p = 0.052) in comparison to -1.00 diopters spherical equivalent. The 95% confidence interval around the mean spherical equivalent in the over-sized group extended from -2.7 to 0.0, just including a value of plano, and the 95% confidence interval around the mean spherical equivalent in the same-size group extended from -1.0 to 0.7, just including with a lower limit of -1 diopter. Thus, despite the overlap in the results of the two graft sizing techniques, their distributions are shifted in the desired direction (Fig 1). Hence, despite the borderline p-value (p = 0.052), the confidential interval confirms statistical significance, and shows the direction and strength of the resulting refractive outcome, thereby supporting the clinical relevance of our findings.

Though clinically important in its findings, our study is limited in certain areas. First, our investigation is retrospective in nature and thus the study has limitations inherent to that of retrospective studies. Secondly, while looking at the results of a single-surgeon has its advantages (such as eliminating bias from differing levels of skill-sets), it would be of interest to see whether the results could be replicated by other surgeons. A larger prospective study, involving a diverse group of surgeons would be warranted.

In summary, based on the results of our study, we found that using a donor graft that is over-sized by 0.25mm resulted in refraction of greater than -1.00 diopters SE. Using a same-size donor graft resulted in refraction of less than -1.00 diopters SE. Keeping these findings in mind, we recommend using an over-sized donor graft in cases where the fellow eye has a myopic refraction. Conversely, a same-size graft should be used in cases where the fellow eye has a hyperopic refraction. Because anisometropia can be a barrier to useful vision for the patient, the surgeon needs to be mindful of graft-size selection at the time of keratoplasty. In addition to the consideration of graft-size selection, the surgeon may need to use a combination of techiques, in order to achieve the desired refractive outcome for the patient[16–22].

## Supporting information

S1 Dataset Data. (TXT)

S1 Fig Spherical Equivalence at 12 months. (XLSX)

S2 Fig Extent of Anisometropia (absolute value of diopters). (XLSX)
